# Pediatric Ethics Guidelines for Hereditary Medullary Thyroid Cancer

**DOI:** 10.1155/2011/847603

**Published:** 2011-02-06

**Authors:** MSara Rosenthal, DouglasS Diekema

**Affiliations:** 1 Program for Bioethics, Department of Internal Medicine, University of Kentucky, Suite K522, 740 South Limestone Street, Lexington, KY 40536-0284, USA; 2 Treuman Katz Center for Pediatric Bioethics, Seattle Children's Hospital, Department of Pediatrics, MPW 12-1, 1100 Olive Way, Suite 1200, Seattle, WA 98101, USA

## Abstract

Hereditary medullary thyroid cancer is an aggressive cancer for which there is no standard effective systemic therapy, but which can be prevented through genetic screening and prophylactic thyroidectomy. Although this cancer accounts for roughly 17% of all pediatric thyroid cancers, a significant percentage of affected families do not "accept" screening, while many gene carriers delay or refuse prophylactic thyroid surgery for their children. Current genetic screening practices in medullary thyroid cancer are inadequate; more than 50% of index patients with hereditary medullary thyroid cancer present with a thyroid mass; up to 75% have distant metastasis. These proposed pediatric ethics guidelines focus on two ethical issues that affect at-risk children: (1) how do we identify at-risk children whose RET-positive relative refuses to disclose that they carry the mutation? (2) How do we protect RET-positive children whose parents refuse prophylactic thyroidectomy?

## 1. Introduction

Medullary thyroid cancer (MTC) is an uncommon type of aggressive thyroid cancer that does not respond to systemic radioactive iodine, an effective treatment for most other types of thyroid cancer. This cancer's aggressive biological behavior also diminishes the effectiveness of surgical therapy, and there is currently no standard effective chemotherapy for this cancer. 

The etiology of MTC is well documented in the literature; it may occur sporadically or present as part of an autosomal dominant inherited disorder. If inherited, MTC is 100% penetrant [1], although the age of onset is variable [1–5]. In 1993, germline mutations in the RET proto-oncogene were found to be responsible for hereditary MTC [3], allowing genetic testing to be used as the primary tool for detecting its presence in at-risk family members. We use the term *hereditary medullary thyroid cancer* to discuss both familial medullary thyroid cancer (FMTC) and medullary thyroid cancer arising from MEN 2 syndromes (MEN 2A and MEN 2B). In the general population, MTC accounts for 5%–8% percent of all thyroid cancers [6–8] and about 15% of all thyroid cancer-related deaths [6]. However, the published statistics on this type of cancer are based on narrow studies and a small literature; the true incidence and prevalence of MTC is likely much higher than what has been reported. According to 2009 SEER data available from the National Cancer Institute, we estimate the national incidence rate of MTC to be approximately 3000 new index cases per year, with a national prevalence of roughly 35,000. Since MTC can be either inherited (familial or hereditary) or not inherited (sporadic), it is also standard of care to offer all index patients genetic testing to rule out the genetic mutation for the inherited form. If an index patient decides not to get tested, he or she may unknowingly represent a large kindred comprising dozens, or hundreds, of at-risk individuals who may eventually die from this cancer because the opportunity for genetic screening and prophylactic surgery was missed. Family members of positive probands will develop medullary thyroid cancer if they, too, test positive for the mutation [1]. 

Roughly 25%–30% of known MTC cases are hereditary [1, 4, 6, 8]. The reported incidence and prevalence rates significantly underestimate the number of hereditary cases, since it does not account for undiagnosed cases in unreported kindreds. It also does not account for the many people who have the gene mutation for MTC, but have not yet developed this cancer. Surgery represents the only effective curative treatment [9], but must occur before the cancer has spread beyond the thyroid gland. This provides the rationale for identifying MTC early through screening. In the absence of early identification through genetic screening, MTC typically presents in a later stage, often as metastatic disease for which there is no effective curative treatment. Chemotherapy and external beam radiation therapy are typically only palliative or ineffective in treating unresectable metastatic MTC. 

In the pediatric population, MTC represents 17% of all childhood thyroid cancers [3], and in children who test positive for the gene (and therefore identified as having hereditary MTC), the American Thyroid Association recommends thyroidectomy at the following age intervals [4]: ages 0-1 for RET mutations that carry the highest risk for aggressive MTC at young ages, classified as "ATA-D"; before the age of five for RET mutations that carry a high risk of aggressive MTC at any age, classified as "ATA-C"; beyond the age of 5 for RET mutations that carry a lower risk of aggressive MTC classified as "ATA-B" or "ATA-A," so long as the child has been carefully evaluated and monitored, and there are no other clinical signs that suggest that MTC has developed 

### 1.1. Genetic Testing

RET genetic mutation testing has been shown to be more sensitive than traditional biochemical screening. In one of the first studies to establish the role of genetic testing, which involved 300 members of four large kindreds, 14 young individuals with RET mutations had normal plasma calcitonin levels, while thyroidectomy revealed small foci of MTC in all eight of these 14 individuals who agreed to undergo surgery [5]. Germline RET mutations have been observed in 3%–9% of MTC patients with no family history of MTC [10–12] indicating that a significant number of apparently sporadic cases of MTC are due to occult or de novo germline RET mutations. As time progresses, more sites of potential mutation in the RET proto-oncogene have been associated with hereditary MTC, resulting in genetic analysis of progressively more exons and suggesting that the rate of unsuspected familial cases is likely much higher. Genetic testing for hereditary MTC syndromes has had an enormous impact on reducing the burden of this disease for families [2] and has the potential to dramatically reduce the incidence of MTC when it occurs as part of these hereditary syndromes. 

Since the discovery of RET mutations, and the availability of genetic testing and screening for these mutations, the vast majority of literature published in this area [2, 3, 13–41] acknowledges the value of genetic screening in at-risk family members and prophylactic thyroidectomy in those who test positive [13, 42]. The current standard of care is to recommend genetic testing for all at-risk family members of the positive proband, assuming the positive proband consents to the release of information to family members. It is standard of care to offer prophylactic therapy to all family members who test positive for the gene mutation and are identified as being part of a hereditary MTC kindred.

## 2. Ethical Issues with RET Mutation Screening

In this unique inherited cancer context, genetic testing remains the only effective route to prevention and treatment for hereditary MTC [9, 13]. Using the "reasonable person standard," a reasonable person who understands and appreciates the benefits of genetic testing for MTC ought to choose to be tested. Yet this is not always the case. Uptake of this testing is significantly less than 100% [10–12, 19, 20, 42–45], indicating that the apparently rational decision is not being made or that there are barriers to understanding and appreciating the benefits of genetic testing. 

Clearly, significant numbers of at-risk individuals are not being tested or notified about MTC, which means that many individuals are not making a reasonable decision about getting tested and many others are failing to notify family members. 

Unfortunately, the uptake of genetic testing among relatives of patients with MTC is inadequate and not well studied. Social and ethical barriers to genetic screening for MTC may include literacy, education, income, culture/religion, and social/family relationships. The positive proband may exercise his or her autonomy and decide not to disclose his/her test results to family members at-risk. In the United States, the Health Insurance Portability and Accountability Act (HIPAA) protects a positive proband's right to privacy, thereby preventing those at-risk from being tested. Although some physicians in these circumstances may choose to breach HIPAA and notify at risk family members, this is not an option that has been ethically or legally resolved [46]. Both the decision to test and the decision to disclose test results to at risk relatives directly impact prevention of this cancer. Even when results are disclosed to at-risk relatives, decisions about prophylactic therapy need to be made. The strained resources of genetic counseling services and accepted clinical practice suggest that genetic counselors are largely unavailable to patients with MTC [17, 18, 47–49]. Even in European countries that offer genetic counseling free of charge, patients report that genetic counseling is inadequate and flawed [50–52]. This suggests a profound misunderstanding of the meaning of genetic test results for this cancer, as well as covert socioethical barriers to screening, including inadequate decision-making capacity, limited access to healthcare, and cultural, religious, and economic factors. There are no specific guidelines regarding genetic screening, disclosure of results, or discussion of results for children with a family history of MTC. 

In the one qualitative study that specifically looked at ethical issues in hereditary MTC, the investigator interviewed members from only one family and reported that genetic counseling was inadequately delivered [18]. This same author [17] also interviewed 21 patients who had thyroidectomies consequent to a hereditary MTC diagnosis and found that the genetic issues were poorly appreciated. A nursing study followed MTC patients on an MTC Listserv and found serious problems with informed decision-making [53]. Brauer et al. [20] observed problems in long-term follow-up data on affected kindreds, suggesting that we know next to nothing about disclosure patterns among probands and how information is communicated or filtered by family members. 

These clinical and psychosocial conditions demonstrate the need to protect vulnerable populations and establish clear ethical guidelines for MTC screening and management in the pediatric population.

In addition to the screening issues, barriers ranging from poor comprehension of the clinical facts to healthcare access may lead at-risk individuals to refuse life-saving prophylactic thyroidectomy for themselves or their children. 

Since genetic screening involves the dissemination of complex information, such barriers can seriously interfere with decisions about testing, disclosure of test results to family members, and decisions affecting children of families at risk. There is no guideline regarding whether assent should be required and, if so, at what age. 

More than 50% of index patients with hereditary MTC present with a thyroid mass, and up to 75% have distant metastasis [19, 42]; this further suggests that genetic screening practices are currently inadequate. Given that MTC accounts for roughly 17% of all pediatric thyroid cancers, the literature reports 15% of affected families that do not "accept" screening [43], and many gene carriers delay or refuse prophylactic thyroid surgery for their children [44]; children who are relatives of index patients are at risk of going undetected. We focus on two ethical issues that affect at-risk children: (1) *how we do identify at-risk children with an RET-positive relative who refuses to disclose that they have the mutation?* (2) *How do we protect RET-positive children whose parents refuse prophylactic thyroidectomy?*

## 3. The Case for Mandated Newborn Screening for RET

There is a strong ethical justification for mandated newborn screening for mutations in the RET gene, particularly when the American Thyroid Association recommends RET mutation testing in children "shortly after birth" in some cases, or before the age of 5 in other cases [4]. RET mutation screening not only meets all of the classical Wilson-Jungner criteria [54] for mandated newborn screening, but a much clearer case can be made for this screening test than some diseases that are already screened for, such as 2-MBG (2-methylbutyryl-coenzyme A dehydrogenase deficiency), for which only a "handful of infants have been diagnosed" with no clear treatment available [55]. Even in PKU, for which screening identifies 200 affected children annually, there are not multiple at-risk family members associated with each positive screen. RET mutation screening in newborns, on the other hand, can identify large kindreds at risk. One positive screen can lead to many more individuals at risk in both pediatric and adult populations. Based on 2007 U.S. birth rate statistics, as well as the latest SEER data on the lifetime risk of developing thyroid cancer, we know that, every year, 36,500 babies are born who will ultimately get thyroid cancer. We know that around 8% of these cancers will be medullary thyroid cancer, meaning that nearly 3000 of the babies born in the USA in 2007 will get medullary thyroid cancer at some point in their lives. Based on these statistics, we safely estimate that roughly 1000 newborns in the USA in 2007 would develop hereditary medullary thyroid cancer, which could be picked up by a newborn screening program. Additionally, during the first 20 years of screening, a newborn screening program has the potential to pick up significant numbers of at risk older children and adults who had not been screened as newborns. For example, if we assume at least 5 unscreened family members at-risk for each of these 1,000 positive newborn screens we could detect annually, we may be able to identify and/or prevent 5,000 cases of MTC annually. 

The ten criteria proposed by Wilson-Jungner [54] have been endorsed by the WHO and the President's Council on Bioethics [55] for the applicability of any newborn screening program.

(1)The condition sought should be an important health problem. 

(2)There should be an accepted treatment for patients with recognized disease.

(3)Facilities for diagnosis and treatment should be available.

(4)There should be a recognizable latent or early symptomatic stage.

(5)There should be a suitable test or examination.

(6)The test should be acceptable to the population.

(7)The natural history of the condition, including development from latent to declared disease, should be adequately understood.

(8)There should be an agreed policy on whom to treat as patients.

(9)The cost of case finding (including diagnosis and treatment of patients diagnosed) should be economically balanced in relation to possible expenditure on medical care as a whole. 

(10)Case-finding should be a continuing process and not a "once and for all" project.

MTC meets all of the above criteria. In the case of criterion 5, the recommendation for newborn screening is reasonable because genetic testing can pinpoint an identifiable RET mutation in 95% of persons with MEN 2 syndromes and in 88% of those with FMTC [1, 4, 56]. In addition, about 1%–9% of apparently sporadic cases will be found to have identifiable RET mutations [4, 56]. Of those individuals found to have an identifiable RET mutation, 100% will develop MTC without any therapeutic intervention, although the age of onset will be variable [1]. Thus, we can prevent MTC in approximately 90% of at-risk individuals with current genetic testing. Furthermore, genetic counseling and testing of family members of mutation-positive cases would lead to identification of entire affected families that otherwise may have gone undetected. Thus, one positive newborn screening test that identifies a RET mutation can lead to prevention of multiple cases of MTC. As with any screening test, the methodology used influences the sensitivity of the test. The vast majority of cases of MEN2 and FMTC are due to mutations affecting cysteine residues in exons 10 and 11 of the RET gene [1, 56]. However, testing only for mutations affecting the cysteine residues would miss cases of MEN2 and FMTC caused by mutations at other sites in the RET gene. While sequencing of the RET gene would detect these additional cases, it would also yield a significant number of variants of unknown clinical significance, which would raise the question of how to follow individuals in whom such variants were detected, since no biochemical test exists to confirm the diagnosis of either MEN2 or FMTC. As with all other newborn screening tests, it would be necessary to explore the most effective way of performing RET gene mutation screening before it is put into practice. 

In the case of criterion 9, in the USA, cancer accounts for $60.9 billion in direct medical costs and $15.5 billion for indirect morbidity costs [57]. Newborn screening would directly reduce the costs associated with late diagnosis of aggressive tumors. In the specific MTC population, the costs associated with treatment of metastatic disease are closest to the costs associated with pancreatic cancer, which is the most expensive cancer cost of the eight cancers evaluated by Chang et al. [57], at an average cost of $7,282 monthly per patient. This high cost is related to the fact that no effective chemotherapy treatments exist for these cancers. Even this may be an underestimate, since treatment for metastatic MTC is currently only available as part of costly clinical trials. 

The President's Council on Bioethics endorsed the original Wilson-Jungner criteria in 2008, highlighting that this classical criteria would mean the disorder for which mandated newborn screening is recommended: "must pose a serious threat to the health of the child, its natural history must be well understood, and timely and effective treatment must be available, so that the intervention as a whole is likely to provide a substantial benefit to the affected child" [55]. The American Council on Medical Genetics endorses mandatory newborn screening by stating: "Societies have an ethical obligation to protect their most vulnerable members, especially if these people cannot protect themselves. Newborns deserve the special protection afforded by mandatory screening for disorders where early diagnosis and treatment favourably affect outcome . The primary purpose of mandatory newborn screening is to benefit the newborn through early treatment" [55]. We believe that RET gene mutation screening rises to this standard.

## 4. Pediatric Ethics Guidelines

Given the clear benefits of early screening and prophylactic treatment and the problems with barriers to informed consent and decision-making in the adult population, we propose that the decision to have children at risk for hereditary MTC tested should be removed from the parents or guardians, who currently frequently elect to decline having their children tested. Instead, we suggest that screening for the RET mutation testing for hereditary MTC ought to be implemented into existing state newborn screening programs and treated similarly to other newborn screening tests, which include the screening for hypothyroidism. Implementation of such guidelines will significantly increase uptake of genetic testing in this population, which in turn could greatly improve thyroid cancer prevention and reduce costs associated with the significant morbidity of this cancer. We further propose ethical guidelines to deal with the older pediatric population to protect them from unnecessary and significant harms resulting from poor parental or guardian decision-making.

### 4.1. Proposed Ethics Guideline 1: Genetic Screening in Pediatric Populations

Outside of the newborn screening context (which allows parents to opt out, but does not explicitly seek consent), genetic screening in pediatric populations requires informed consent from a child's surrogate decision-maker (parent or guardian). The incidence of parents refusing to consent to medical procedures or treatment for their children in a number of pediatric clinical scenarios is well documented [58–60], as are the numerous barriers to informed consent for complex genetic syndromes. Beneficence-based obligations support mandatory newborn screening for RET proto-oncogene mutations. RET mutation screening meets the ethical criteria for newborn screening proposed by the President's Council on Bioethics [55] and the traditional Wilson-Jungner criteria [54]. Newborn screening removes decision-making from the surrogate, which is frequently a barrier to screening in the neonatal population. For screening of older children, consent from the legally authorized decision-maker would be necessary for screening, but this would be phased out over time as newborn screening was universally adopted. Parental refusal to allow screening may require implementing the recommendation outlined in Proposed Ethics Guideline 3, depending on the risk of aggressive MTC developing in childhood, as in situations where the RET mutation is classified as "ATA-D" [4].

### 4.2. Proposed Ethics Guideline 2: Disclosure of RET Mutation

As with newborn screening, positive results should be shared with a child's physician and with the child's legally authorized decision-makers. Children over the age of 14 should be included in the disclosure of this information unless a strong reason exists for excluding them. To mitigate potential problems with surrogate decision-makers misinforming an older child, or deciding to withhold the information from an older child, disclosure of results should be provided in a family conference that includes, if possible, at least three of the following experts: a pediatric endocrinologist, surgeon or oncologist; genetic counselor, social worker, and ethicist. The disclosure of results may present complex issues for the family and should be done using a multidisciplinary team approach.

### 4.3. Proposed Ethics Guideline 3: Surrogate's Refusal of Prophylactic Thyroidectomy

In patients where prophylactic thyroidectomy is strongly recommended according to clinical practice guidelines [4], surrogates who refuse such surgery for their children should be assessed for their understanding and appreciation of the issues, including their rationale for the refusal. The physician has an obligation to exhaust efforts to explain and disclose the clinical situation, including weighing the benefits and risks of delaying thyroidectomy and using other biochemical markers in children with RET mutations that carry lower risks, such as those classified as "ATA-B" and "ATA-A" [4]. In higher-risk scenarios, such as mutations classified as "ATA-D" [4], every effort should be made to properly inform and seek permission from the legally authorized decision-maker, and an ethics consultation should be obtained. If refusal persists, and harm to the patient is imminent and foreseeable, beneficent-based moral obligations require that the practitioner, with the support of the ethics and legal consultant, seek state intervention through a court order or the involvement of child protective services. We believe these cases invoke the Harm Principle [59] as justification for seeking state intervention when parents refuse life-saving medical treatment (thyroidectomy) that carries a good likelihood of preventing significant harm (i.e., death from thyroid cancer). The Harm Principle, originally outlined by J. S. Mill, in his On Liberty treatise (1859) states: 

"The only purpose for which power can be rightfully exercised over any member of a civilized community, against his will, is to prevent harm to others... The only part of the conduct of any one, for which (an individual) is amenable to society, is that which concerns others. In the part which merely concerns himself, his independence is, of right, absolute. Over himself, over his own body and mind, the individual is sovereign." 

The Harm Principle establishes that a competent individual has complete autonomy over his/her own beliefs and actions so long as those beliefs or actions do not create a significant likelihood of serious harm to another person. If one's actions or decisions place another in harm's way, state intervention is justified. In cases where prophylactic surgery can be delayed until the age of consent, aggressive follow-up of the patient should include repeated attempts to discuss prophylactic surgery with the parent or guardian. In cases where delay of thyroidectomy is likely to result in disseminated cancer, however, state intervention should be sought to overturn a parental decision to refuse thyroidectomy.

### 4.4. Proposed Ethics Guideline 4: Disclosure of Risk and the Duty to Warn

In cases where probands have not disclosed their RET mutation status to their at-risk relatives, the patient should always be asked permission to disclose. If permission is denied, an ethics consult should be obtained, and the patient's reasons for refusing permission should be explored. The patient should also be provided with the option of disclosure without being directly identified. In cases where refusal of disclosure puts the at-risk relative(s) in danger of imminent serious harm, the physician must recognize that there are limits to patient autonomy and that confidentiality "ends where public peril begins" [61]. The ethical justification and moral imperative for notifying at-risk relatives against patients' wishes is supported by "duty to warn" case law precedents [46], as well as the American Society of Human Genetics Social Issues and Subcommittee on Familial Disclosure [62]. Known at-risk relatives should be notified of the risk without specifically identifying the patient, and privacy laws should be modified to allow for this. In cases where harm to minors is imminent, implementation of Ethics Guideline 3 may be warranted.

## 5. Conclusions

Hereditary MTC is a different clinical context than other hereditary cancer syndromes (e.g., BRCA1, BRCA2), where penetrance is significantly lower, the genetic test can yield ambiguous results, and the effectiveness and perceived benefits of "treatment" is unknown or has significant side effects. Even with full understanding and appreciation of risk profile, a range of socioeconomic factors may interfere with one's ability or willingness to obtain genetic testing, including access, counseling, socioeconomic status and location. Other factors that may interfere with disclosure of test results to at-risk family members include awareness of family members in situations of half-siblings and adoption, or communication barriers among family members due to dysfunction or estrangement. 

Hereditary MTC is the only autosomal dominant cancer with 100% penetrance where genetic screening is the singular pathway to prevention and/or cure, which includes a treatment without significant morbidity. Suspected socioethical barriers to genetic screening and prophylactic therapy include inadequate genetic counseling and problems related to understanding of genetics or risk assessment [47–49, 63, 64]; decision-making capacity problems [65], which may involve literacy, numeracy, and education levels; access to healthcare, which may involve income and insurance coverage [45]; family and cultural dynamics [66]; confidentiality [46, 67]; religious and cultural beliefs; social and community location. Since genetic screening involves the dissemination of complex information, such barriers can seriously interfere with decisions about testing, disclosure of test results to family members, and decisions about prophylactic surgery. At-risk children should be protected from preventable harms associated with metastatic MTC, resulting from either inadequate or irrational decisions made by their guardians. These proposed pediatric ethics guidelines seek to mitigate harms associated with hereditary MTC in the pediatric population.
